# Environmental Drivers of *Ganoderma resinaceum* and *Ganoderma enigmaticum* Mycelial Growth on Malted Sorghum and Associated Changes in Antioxidant Activity and Mineral Composition: Implications for Pito Bio‐Enrichment

**DOI:** 10.1002/fsn3.72276

**Published:** 2026-08-20

**Authors:** Florence Adwoa Ayirezang, William Otoo Ellis, Ibok Nsa Oduro, Matilda Dzomeku, Gideon Adotey

**Affiliations:** ^1^ Science Laboratory Technology Department Accra Technical University Ghana; ^2^ Food Science and Technology Department Kwame Nkrumah University of Science and Technology Kumasi Ghana; ^3^ CSIR‐Food Research Institute Accra Ghana

**Keywords:** DPPH assay, food‐grade substrate, functional beverage, fungal biomass, inoculum size, solid‐state cultivation

## Abstract

Ganoderma species are increasingly recognized as promising sources of bioactive compounds for food and nutraceutical applications. This study evaluated the effects of temperature, light regime, and inoculum size on the mycelial growth of *Ganoderma resinaceum* and *Ganoderma enigmaticum* cultivated on malted sorghum, with implications for future pito bio‐enrichment. A factorial design was used with temperatures of 25°C, 30°C, and 35°C, light conditions of complete darkness or a 12 h light/12 h dark cycle, and inoculum sizes of 2%, 4%, 6%, and 8%. Significant treatment effects on mycelial length were observed for both species at all incubation days (*p* < 0.001). Mycelial growth was fastest at 30°C in the dark with 6% to 8% inoculum; by day 6, GE308D and GR308D had attained the maximum mycelial length of 8.7 mm. GE308D (53.00% ± 0.10%) and GR308D (49.00% ± 0.10%) had the largest mycelial masses. GR308D showed the strongest antioxidant activity (EC50 6.7 ± 0.10 mg/mL), whereas GE302D showed the weakest (13.6 ± 0.11 mg/mL). Potassium was highest in GR254D (472.00 ± 17.00 mg/kg), phosphorus in GE308D (940.67 ± 21.50 mg/kg), magnesium in GR308D (4004.00 ± 92.00 mg/kg), calcium in GE308D (884.00 ± 16.00 mg/kg), and sulfur in GR308D (213.00 ± 15.00 mg/kg). These findings identify 30°C, darkness, and 6% to 8% inoculum as favorable conditions for biomass production, enhanced antioxidant activity and mineral enrichment.

## Graphical abstract

Cultivation of *Ganoderma resinaceum* and *Ganoderma enigmaticum* on malted sorghum at 30°C in the dark with a 6% to 8% inoculum led to accelerated mycelial colonization, increased biomass yield, greater antioxidant activity, and improved mineral enrichment, indicating potential for pito bio‐enrichment.

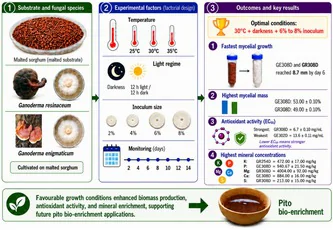

## 1 Introduction

Mushrooms have long been valued for their nutritional, medicinal, and industrial applications, with species of the genus *Ganoderma* widely recognized for their bioactive properties (Mizuno and Nishitani 2013; Wang et al. 2020). Among these, *Ganoderma resinaceum* and *Ganoderma enigmaticum* have recently been identified in Ghana (Adotey et al. 2023), presenting an opportunity for their integration into traditional food systems. Pito, an alcoholic beverage made from fermented sorghum, is very important both culturally and economically in West Africa, especially in Ghana. The production of pito may benefit from the use of mycelial extracts or biomass from Ganoderma species, given the growing interest in functional food applications. However, attaining scalable production and guaranteeing the effective use of these species in fermentation‐based food products require improving mycelial growth under regulated environmental conditions.

Important environmental factors that affect mycelial density, the synthesis of bioactive compounds, and total biomass yield include temperature, light exposure, and inoculum size. These variables also affect the growth and metabolic activity of *Ganoderma* species. Exact temperature control improves enzymatic activity and substrate colonization rates, according to earlier research on *Ganoderma lucidum* (Song et al. 2007; Jayasinghe et al. 2008; Du et al. 2019). It has also been demonstrated that light conditions control the production of secondary metabolites and mycelial differentiation (Amano et al. 2007; Wang et al. 2011; Fabros et al. 2024). Despite this wealth of knowledge on other Ganoderma species, little is known about the specific growth requirements of *G. resinaceum* and *G. enigmaticum*, particularly in their potential use in traditional African food and beverage fermentation.

There is a compelling argument to investigate using *Ganoderma* mycelium in pito production in Ghana due to the growing demand for functional foods. Microbial interactions are used in fermented drinks, such as pito, to improve flavor, nutritional content, and shelf life. By adding *G. resinaceum* and *G. enigmaticum* to the brewing process, bioactive compounds with possible health benefits could be added, and product innovation could be aided. However, creating optimal growth conditions to optimize mycelial biomass production is essential to the application's feasibility.

This study investigated the effects of temperature, light exposure, and inoculum size on the mycelial growth of *Ganoderma resinaceum* and *Ganoderma enigmaticum* to determine their potential suitability for future pito production. By elucidating the ideal cultivation parameters, this research seeks to provide foundational knowledge for the controlled propagation of these species, ultimately contributing to their sustainable application in Ghana's functional food and brewery industries.

## 2 Materials and Methods

### 2.1 Experimental Design

A factorial experimental design was employed to evaluate the effects of temperature, light conditions, and inoculum size on the mycelial growth of *G. resinaceum* and *G. enigmaticum* on malted sorghum under controlled laboratory conditions. This approach enabled the assessment of both individual (main) and interactive effects of these factors.

Mycelial growth was evaluated under a factorial combination of three temperature levels (25°C, 30°C, and 35°C), two light regimes (complete darkness and a 12‐h light/12‐h dark cycle), and four inoculum sizes (2%, 4%, 6%, and 8%). Each environmental condition was replicated, and measurements were taken at seven time points (2, 4, 6, 8, 10, 12, and 14 days) to monitor the progression of mycelial development.

### 2.2 Sample Collection and Preparation

Young red sorghum (chere) was purchased at the farm gate in Jirapa, Upper West Region, Ghana, as described previously (Ayirezang et al. 2025). *G. enigmaticum* and *G. resinaceum* isolates originated from the Volta Basin, where identity and UPLC‐QTOF‐MS metabolomic profiles have been established (Adotey et al. 2023). Culture handling, purity checks, and ITS rDNA confirmation followed our earlier protocol; the sequences matched published records (Ayirezang et al. 2025). Strains were maintained on malt extract agar slants at 4°C and subcultured every 3 months, as previously described (Ayirezang et al. 2025).

### 2.3 Sorghum Malt Preparation

The sorghum was sorted for wholesome grains before the malting process; the sorghum grains were initially washed under running tap water and thermally treated in hot water at 85°C for 1 min. They were soaked in distilled water for 24 h, with the water being refreshed every 12 h. After soaking, the grains were drained for an hour before being evenly spread on hydroponic trays and covered with a moist cotton napkin to maintain humidity. The grains were moistened with distilled water and kept in a growth room for 3 days, with morning and evening monitoring to facilitate sprouting. The growth room maintained humidity levels between 42.3% and 50.5%, temperatures from 24.6°C to 30.2°C, and light intensity ranging from 110.00 lx to 143.00 lx using the Samsung galaxy sensor mobile application. After 3 days of germination, the sprouted grains were harvested and sun‐dried in a mica sheet solar dryer at 45°C to 50°C for 3 days (Ayirezang et al. 2025).

### 2.4 Media Preparation

Malt extract agar was prepared according to the manufacturer's protocol. The media was allowed to cool to approximately 45°C—50°C, at which point 5 mL of Gentamicin was added. After mixing the mixture well, it was carefully poured into sterile petri dishes to avoid air bubbles and allowed to set.

### 2.5 Mushroom Reactivation

For aseptic reactivation, a small portion of the mushroom culture was cut into three smaller segments. Each segment was then placed on the surface of the malt extract agar in sterile Petri dishes, ensuring a distance of approximately 3.0 cm–4.5 cm between each placement. The inoculated cultures were subsequently wrapped in a sterile transparent zip‐lock bag, transported aseptically to the growth room, and incubated at 30°C for 7 days (Ayirezang et al. 2025).

### 2.6 Mycelial Growth of Mushroom on Malted Sorghum

Autoclaved sorghum samples at 121°C for an hour were aseptically inoculated with reactivated *Ganoderma* cultures (Ayirezang et al. 2025) and were subjected to the various environmental conditions earlier stated. Linear mycelial growth was measured every 2 days over the 14‐day experimental period. Growth was assessed by measuring the radial expansion of the mycelium on the substrate (malted sorghum) in millimeters using a transparent ruler. On the 14th day, the mycelial mass of each sample was harvested and dried at 45°C ± 5°C until a constant weight was achieved, and the dry weight was recorded to determine biomass production.

### 2.7 Sample Coding and Experimental Treatments

All treatment samples were assigned unique alphanumeric codes representing species identity, incubation temperature, inoculum size, and light condition. The first two letters denote the *Ganoderma* species (GR for *G. resinaceum* and GE *for G. enigmaticum*), followed by a two‐digit number indicating the incubation temperature in °C (for example: 25, 30, or 35). This is followed by a single digit representing the inoculum size percentage (2%, 4%, 6%, or 8%) and a final alphabet (D for darkness or L for 12‐h light/dark cycle).

For example, the code “GR308D” refers to *Ganoderma resinaceum* incubated at 30°C with an 8% inoculum size under complete darkness. Table 1 provides full treatment descriptions corresponding to each code.

**TABLE 1:** Sample code key for Ganoderma treatments.

Sample code	Species	Temperature (°C)	Inoculum size (%)	Light condition
GE252D	*G. enigmaticum*	25	2	Darkness
GE254D	*G. enigmaticum*	25	4	Darkness
GE256D	*G. enigmaticum*	25	6	Darkness
GE258D	*G. enigmaticum*	25	8	Darkness
GE252L	*G. enigmaticum*	25	2	12 h light/12 h dark
GE254L	*G. enigmaticum*	25	4	12 h light/12 h dark
GE256L	*G. enigmaticum*	25	6	12 h light/12 h dark
GE258L	*G. enigmaticum*	25	8	12 h light/12 h dark
GE302D	*G. enigmaticum*	30	2	Darkness
GE304D	*G. enigmaticum*	30	4	Darkness
GE306D	*G. enigmaticum*	30	6	Darkness
GE308D	*G. enigmaticum*	30	8	Darkness
GE302L	*G. enigmaticum*	30	2	12 h light/12 h dark
GE304L	*G. enigmaticum*	30	4	12 h light/12 h dark
GE306L	*G. enigmaticum*	30	6	12 h light/12 h dark
GE308L	*G. enigmaticum*	30	8	12 h light/12 h dark
GE352D	*G. enigmaticum*	35	2	Darkness
GE354D	*G. enigmaticum*	35	4	Darkness
GE356D	*G. enigmaticum*	35	6	Darkness
GE358D	*G. enigmaticum*	35	8	Darkness
GE352L	*G. enigmaticum*	35	2	12 h light/12 h dark
GE354L	*G. enigmaticum*	35	4	12 h light/12 h dark
GE356L	*G. enigmaticum*	35	6	12 h light/12 h dark
GE358L	*G. enigmaticum*	35	8	12 h light/12 h dark
GR252D	*G. resinaceum*	25	2	Darkness
GR254D	*G. resinaceum*	25	4	Darkness
GR256D	*G. resinaceum*	25	6	Darkness
GR258D	*G. resinaceum*	25	8	Darkness
GR252L	*G. resinaceum*	25	2	12 h light/12 h dark
GR254L	*G. resinaceum*	25	4	12 h light/12 h dark
GR256L	*G. resinaceum*	25	6	12 h light/12 h dark
GR258L	*G. resinaceum*	25	8	12 h light/12 h dark
GR302D	*G. resinaceum*	30	2	Darkness
GR304D	*G. resinaceum*	30	4	Darkness
GR306D	*G. resinaceum*	30	6	Darkness
GR308D	*G. resinaceum*	30	8	Darkness
GR302L	*G. resinaceum*	30	2	12 h light/12 h dark
GR304L	*G. resinaceum*	30	4	12 h light/12 h dark
GR306L	*G. resinaceum*	30	6	12 h light/12 h dark
GR308L	*G. resinaceum*	30	8	12 h light/12 h dark
GR352D	*G. resinaceum*	35	2	Darkness
GR354D	*G. resinaceum*	35	4	Darkness
GR356D	*G. resinaceum*	35	6	Darkness
GR358D	*G. resinaceum*	35	8	Darkness
GR352L	*G. resinaceum*	35	2	12 h light/12 h dark
GR354L	*G. resinaceum*	35	4	12 h light/12 h dark
GR356L	*G. resinaceum*	35	6	12 h light/12 h dark
GR358L	*G. resinaceum*	35	8	12 h light/12 h dark

### 2.8 Chemical Analyses

Based on the growth conditions and the mycelium mass percentage (≥ 25%), some combined parameters were subjected to further analysis such as proximate analysis, antioxidant activity, and mineral analysis.

### 2.9 Proximate Analysis

#### 2.9.1 Moisture Content Determination

The moisture content of the samples was determined using the AOAC (2005) method. The weight of an empty dish was first observed (Y), and then around 10 g of the sample was added; the total weight was then recorded (X). After 5 h of heating the sample‐containing dish to 105°C, it was allowed to cool to ambient temperature in a desiccator before being weighed again (Z). The moisture content was calculated based on the weight difference before and after drying.

#### 2.9.2 Ash Content Determination

The ash content of the samples was determined using the AOAC (2005) method. After first recording the weight of an empty crucible (Y), 10 g of the sample was added to the crucible, and the total weight was recorded as Z. After that, the crucible and its contents were heated for 18 h at 600°C in a furnace. The heated crucible was carefully moved to a desiccator to cool after heating, avoiding the absorption of moisture. The crucible and ash residue's final weight after cooling was noted (X). The ash content was calculated based on the weight difference before and after heating.

#### 2.9.3 Determination of Crude Fiber

Crude fiber was determined following Pearson's Composition and Analysis of Foods (Kirk and Sawyer 1991). The crude fiber content was calculated from the difference between the mass of the dry insoluble residue and the mass of the ash. The sample was boiled in 200 mL of 0.255 N H_2_SO_4_ for 30 min and filtered through pre‐weighed, pre‐dried filter paper. The residue was returned to the original flask, boiled in 200 mL of 0.313 N NaOH for 30 min, and filtered again onto the same paper. The paper plus residue was dried at 100°C for 30 min, cooled in a desiccator, and weighed, then ashed at 550°C for 4 h and reweighed. Crude fiber was calculated as the mass of dried insoluble residue minus the mass of ash.

#### 2.9.4 Fat Determination

Fat content was determined according to AOAC (2005). An empty crucible was weighed, then 10 g of the sample was weighed. A cotton‐lined extraction thimble was weighed, loaded with the sample, and placed in a solvent extraction apparatus containing petroleum ether maintained at 50°C for 1 h. After extraction, the thimble was removed, allowed to dry at room temperature to eliminate residual solvent, and reweighed. Fat content was calculated as the difference between the sample mass before extraction and the mass after extraction, expressed as a percentage of the initial sample mass.

#### 2.9.5 Protein Determination

Crude protein was determined by the Kjeldahl method following AOAC (2005). A 1 g moisture‐free sample was digested with 20 mL concentrated H_2_SO_4_ in a block digester until the solution changed from black to greenish blue. Digestion was accelerated with a catalyst mixture of K_2_SO_4_, CuSO_4_, and selenium powder to convert organic nitrogen to ammonium sulfate. The cooled digest was made alkaline and steam‐distilled to liberate ammonia, which was captured in boric acid containing Tashiro indicator. The distillate was titrated with 0.01 N HCl to a green endpoint. Percentage nitrogen was calculated from the titration values, and crude protein was obtained as %N multiplied by 6.25.

#### 2.9.6 Carbohydrate Determination

The carbohydrate content was estimated based on the AOAC (2005) method. The percentages of moisture, protein, ash, fat, and crude fiber were summed, and their total was subtracted from 100% to determine the carbohydrate content. The calculation was performed by subtracting the sum of these components from 100%.

### 2.10 Antioxidant Activity (DPPH Assay)

Antioxidant activity was measured using the free radical scavenging method described by Languon et al. (2018). This experiment utilized the reagent 2,2‐Diphenyl‐2‐picrylhydrazyl (DPPH). Sample stock concentrations were prepared at 20 mg/mL in distilled water. Two‐fold serial dilutions were performed to obtain seven (7) concentrations (20, 10, 5, 2.5, 1.25, 0.625, and 0.3125 mg/mL). An aliquot of 0.5 mM methanolic DPPH (TCI Chemicals, Tokyo, Japan) solution was added to equal volumes of different concentrations of each sample (concentration range 0–20 mg/mL) and incubated for 20 min in the dark. Assays were performed in triplicate. Absorbance readings were taken using a microplate reader (Tecan Infinite M200 PRO, Switzerland) at a wavelength of 517 nm. Ascorbic acid was used as a standard, and the diluents (water) were used as blanks. The percentage antioxidant activity of the extracts was determined using the following formula:

$$\%\text{Antioxidant activity}=\frac{\text{Absorbance of control}-\text{Absorbance of sample}}{\text{Absorbance of control}}\times 100$$

The effective concentrations required to scavenge 50% of DPPH radicals (i.e., EC_50_ values) were calculated from the graph. The lower the EC_50_, the less the concentration of a drug is required to produce 50% of the maximum effect.

### 2.11 X‐Ray Fluorescence (XRF) Mineral Analysis

About 4 g of the powdered sample (< 100 um) and 0.9 g of the wax binder were weighed using the balance. The two were put together in the mixing container and homogenized using the mill. The mixture was then poured into the die and pressed with 15 tons to a 32 mm pellet using the hydraulic press. The pellet was analyzed using a Vanta VMR XRF spectrometer. Based on the quantity of energy received, the electrons in the samples go to an excitation state, determining the energy level (*n* = 1, *n* = 2, *n* = 3…, etc.). However, once the energy is exhausted, the electrons must collapse back to the ground state, where they emit radiation that identifies the type of mineral inside the samples (Takahashi 2015).

### 2.12 Statistical Analysis

All experimental data were analyzed using ANOVA in Minitab 21.1 software to determine the significance of individual and interactive effects of temperature, light, and inoculum size on mycelial growth. Where significant differences were detected, treatment means were separated using Tukey's post hoc test at *p* < 0.05.

## 3 Results

### 3.1 Growth Phases of *Ganoderma enigmaticum* and *Ganoderma resinaceum*

**FIGURE 1:**
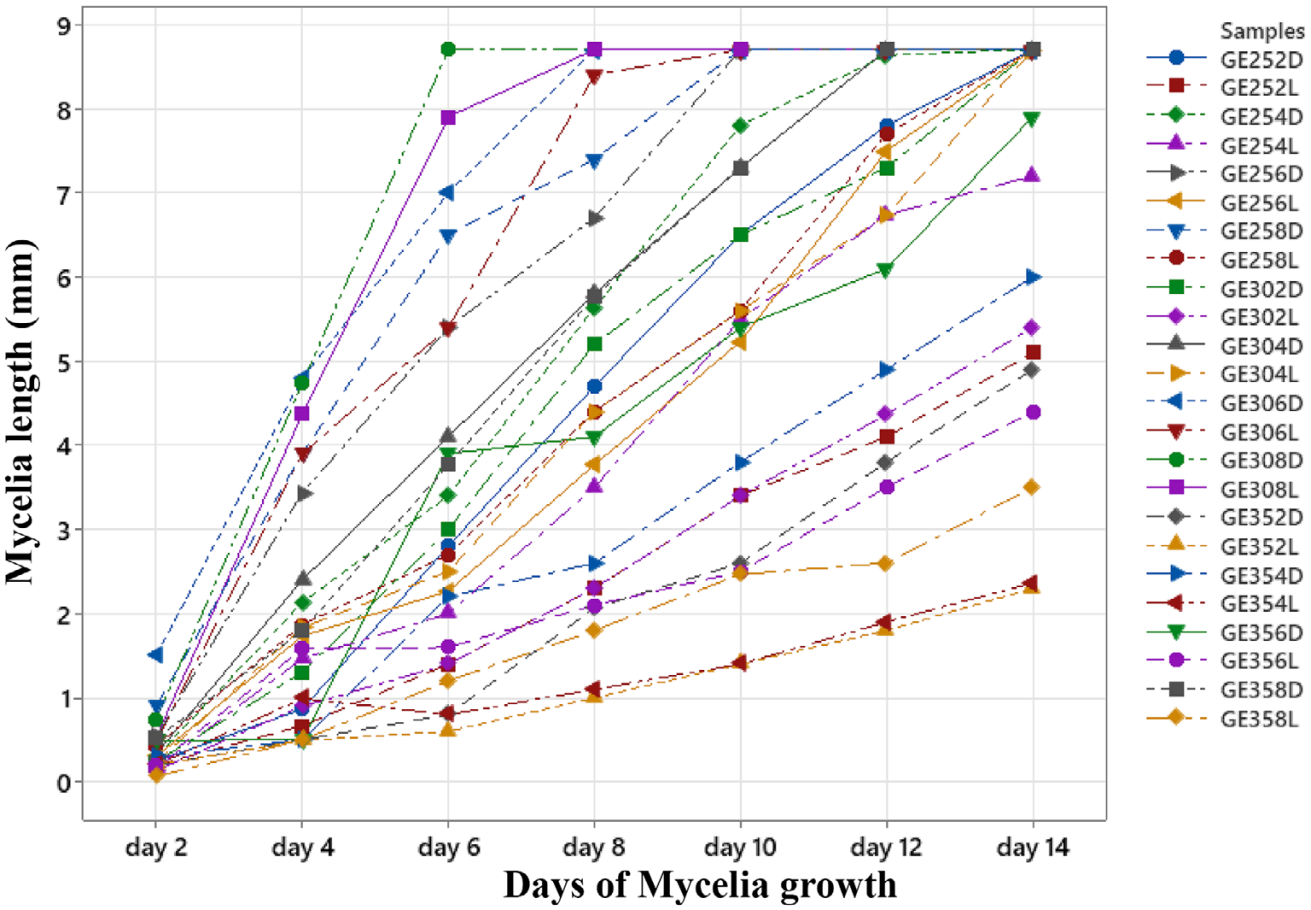
Mycelial length of coded *Ganoderma enigmaticum* for 14 days of incubation. Sample codes reflect species, incubation temperature, inoculum size, and light conditions. See Table 1 for full sample descriptions.

**FIGURE 2:**
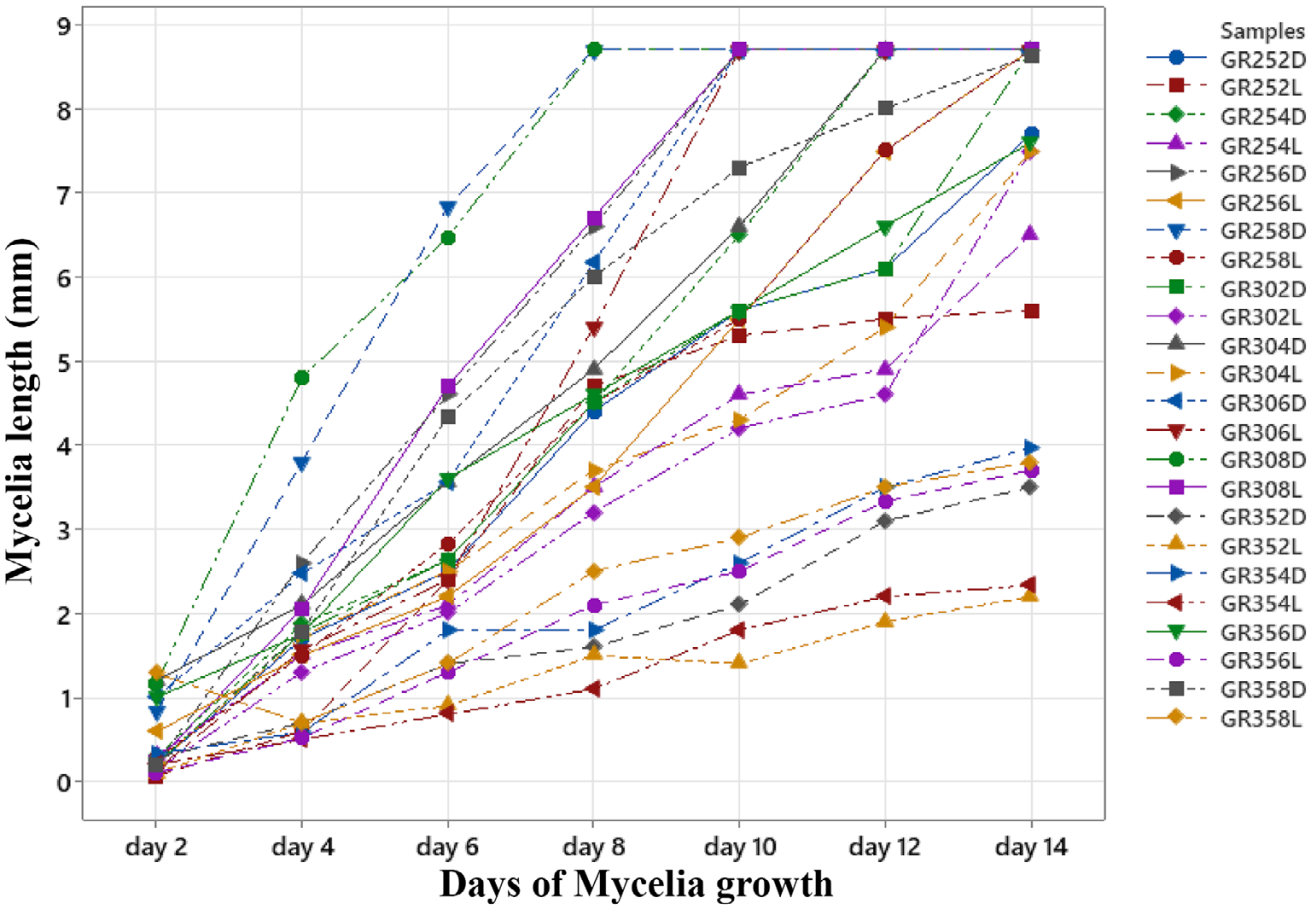
Mycelial length (mm) of coded (*Ganoderma resinaceum*) for 14 days of incubation. Sample codes reflect species, incubation temperature, inoculum size, and light conditions. See Table 1 for full sample descriptions.

Figures 1 and 2 present the mean mycelial growth of *G. enigmaticum* and *G. resinaceum* over a 14‐day incubation period under different environmental conditions. Significant treatment effects (Table 2) on mycelial length were observed for both species throughout incubation (*p* < 0.001).

**TABLE 2:** F‐values and *p*‐values for mycelial length of *Ganoderma enigmaticum and Ganoderma resinaceum acr*oss incubation days.

Day	*G. enigmaticum* F‐value	*G. enigmaticum p*‐value	*G. resinaceum* F‐value	*G. resinaceum p*‐value
Day 2	11.29	< 0.001	12.25	< 0.001
Day 4	66.06	< 0.001	53.27	< 0.001
Day 6	248.81	< 0.001	58.50	< 0.001
Day 8	368.15	< 0.001	313.87	< 0.001
Day 10	562.02	< 0.001	357.37	< 0.001
Day 12	392.69	< 0.001	548.05	< 0.001
Day 14	688.59	< 0.001	302.59	< 0.001

*Note:* ANOVA showed significant differences in mycelial length among treatments for both *G. enigmaticum* and *G. resinaceum* at all incubation days.

*G. enigmaticum* and *G. resinaceum* exhibited three distinct growth phases: a lag phase, an exponential phase, and a stationary phase. From day 2 to day 4, both species experienced a lag phase marked by relatively slow mycelial extension. In this phase, *G. enigmaticum* reached mean mycelial lengths ranging from 0.2 mm to 1.5 mm, while *G. resinaceum* remained below 2 mm. The exponential phase, which lasted from day 4 to day 8, was marked by fast growth, especially in samples with larger inoculum sizes (6% and 8%), which were incubated at 30°C. By days 6 and 8, these samples, including GE308D and GR308D, had grown to the maximum mycelial length ever measured, which was 8.7 mm. On the other hand, samples at 25°C showed a similar pattern, albeit a little later, plateauing between days 10 and 12. At 35°C, mycelial development was significantly slower, with final lengths between 4.0 and 7.0 mm observed by days 12 to 14.

The influence of inoculum size was evident across all treatments. Higher inoculum levels (6% and 8%) resulted in faster colonization and early onset of the stationary phase. Samples like GE308D and GR308D, which combined high inoculum size with optimal temperature (30°C) and darkness, reached maximum growth by day 6, the earliest among all treatments. Other high‐performing treatments, including GE306D, GE258D, GR306D, and GR258D, also reached their maximum recorded mycelial length by day 8, indicating accelerated colonization under favorable temperature, darkness, and higher inoculum conditions. In contrast, lower inoculum sizes (2% and 4%) showed delayed growth, plateauing between days 10 and 14 and maximum lengths ranging from 5.5 to 7.5 mm.

Mycelial development was significantly influenced by light conditions as well. Compared to samples exposed to a 12‐h light/dark cycle, those incubated in total darkness showed somewhat higher and faster mycelial growth. Under dark conditions, GE308D, GE306D, and GE258D for *G. enigmaticum*, and GR308D, GR306D, and GR258D for *G. resinaceum*, reached the maximum recorded mycelial length of 8.7 mm earlier than their corresponding light‐exposed treatments. While samples like GE308L and GR308L eventually reached similar growth levels, they did so more slowly, typically between days 8 and 10.

The stationary phase was evident in high‐performing samples, particularly those grown at 30°C and 25°C with 6% and 8% inoculum sizes. GE308D and GR308D plateaued as early as day 6, while others followed by days 8 to 10. Interestingly, samples grown at 35°C plateaued earlier but reached lower maximum growth values, suggesting possible thermal stress limiting their development. Several samples plateaued at 8.7 mm for multiple days, suggesting that visible mycelial growth had reached the bottom of the substrate container, with potential internal branching occurring within the medium.

Treatment effects on mycelial length were significant for both *G. resinaceum* and *G. enigmaticum* at all incubation days (*p* < 0.001), as shown in Table 2. The F‐value for *G. enigmaticum* rose from 11.29 on day 2 to 688.59 on day 14, indicating that differences between treatments became more pronounced over time. Significant treatment effects were also observed for *G. resinaceum* throughout the incubation period, with day 12 showing the highest F‐value (F = 548.05). These results show that mycelial development varied significantly with temperature, light, and inoculum size, especially during the later growth phases.

### 3.2 Mycelia Mass of *Ganoderma enigmaticum* and *Ganoderma resinaceum*

Table 3 presents the mycelial mass accumulation of *G. enigmaticum* and *G. resinaceum* under different conditions. The highest mycelial mass was recorded at 30°C under dark conditions with an 8% inoculum size, where *G. enigmaticum* reached 53.00% ± 0.10% and *G. resinaceum* reached 49.00% ± 0.10%. Significant differences (*p* ≤ 0.05) were observed between 30°C and 35°C, as growth declined at 35°C. Dark conditions resulted in significantly higher mycelial mass (*p* = 0.039) than the 12‐h light/dark cycle.

**TABLE 3:** Mean mycelia mass (%) of *Ganoderma* spp. after 14 days incubation.

Samples	Mycelia mass (%)
GE 252D	10.67 ± 0.10 ^LMN^
GE 254D	25.00 ± 0.10 ^F^
GE 256D	30.00 ± 0.10 ^E^
GE 258D	30.00 ± 0.60 ^E^
GE 252 L	11.00 ± 0.40 ^OPQ^
GE 254 L	12.50 ± 0.10 ^NOP^
GE 256 L	14.00 ± 0.10 ^MNO^
GE 258 L	23.00 ± 0.10 ^FGH^
GE 302D	26.00 ± 0.10 ^D^
GE 304D	29.00 ± 0.10 ^E^
GE 306D	47.00 ± 0.10 ^bc^
GE 308D	53.00 ± 0.10 ^A^
GE 302 L	10.83 ± 0.66 ^OPQ^
GE 304 L	12.50 ± 0.10 ^NOP^
GE 306 L	14.00 ± 0.10 ^MNO^
GE 308 L	23.00 ± 0.10 ^FGH^
GE 352D	4.00 ± 0.10 ^S^
GE 354D	6.00 ± 0.10 ^RS^
GE 356D	18.00 ± 0.10 ^JKL^
GE 358D	21.00 ± 0.10 ^FGHIJ^
GE 352 L	12.50 ± 0.10 ^NOP^
GE 354 L	14.00 ± 0.10 ^MNO^
GE 356 L	14.00 ± 0.10 ^MNO^
GE 358 L	14.00 ± 0.10 ^MNO^
GR 252D	14.83 ± 0.10 ^LMN^
GR 254D	29.00 ± 0.10 ^E^
GR 256D	31.33 ± 0.10 ^E^
GR 258D	36.00 ± 0.10 ^D^
GR 252 L	4.00 ± 0.10 ^S^
GR 254 L	8.33 ± 0.10 ^QR^
GR 256 L	15.33 ± 0.10 ^LMN^
GR 258 L	21.67 ± 0.10 ^FGHI^
GR 302D	23.83 ± 0.10 ^FG^
GR 304D	44.00 ± 4.10 ^C^
GR 306D	45.00 ± 0.10 ^C^
GR 308D	49.00 ± 0.10 ^B^
GR 302 L	4.00 ± 0.10 ^S^
GR 304 L	9.50 ± 0.10 ^PQ^
GR 306 L	11.00 ± 0.10 ^OPQ^
GR 308 L	19.00 ± 0.10 ^IJK^
GR 352D	15.00 ± 0.10 ^LMN^
GR 354D	16.00 ± 2.10 ^KLM^
GR 356D	20.00 ± 0.10 ^HIJ^
GR 358D	21.70 ± 0.10 ^FGHI^
GR 352 L	3.33 ± 0.10 ^S^
GR 354 L	5.00 ± 0.10 ^RS^
GR 356 L	15.00 ± 0.10 ^LMN^
GR 358 L	15.67 ± 0.59 ^KLMN^

*Note:* Data was reported as means ± standard deviation of mycelia mass of *Ganoderma enigmaticum* and *Ganoderma resinaceum*. Means that do not share the same letter are significantly different (*p* ≤ 0.05). Analysis was performed in triplicates.

### 3.3 Effect of Inoculum Size on Mycelial Biomass Production

**FIGURE 3:**
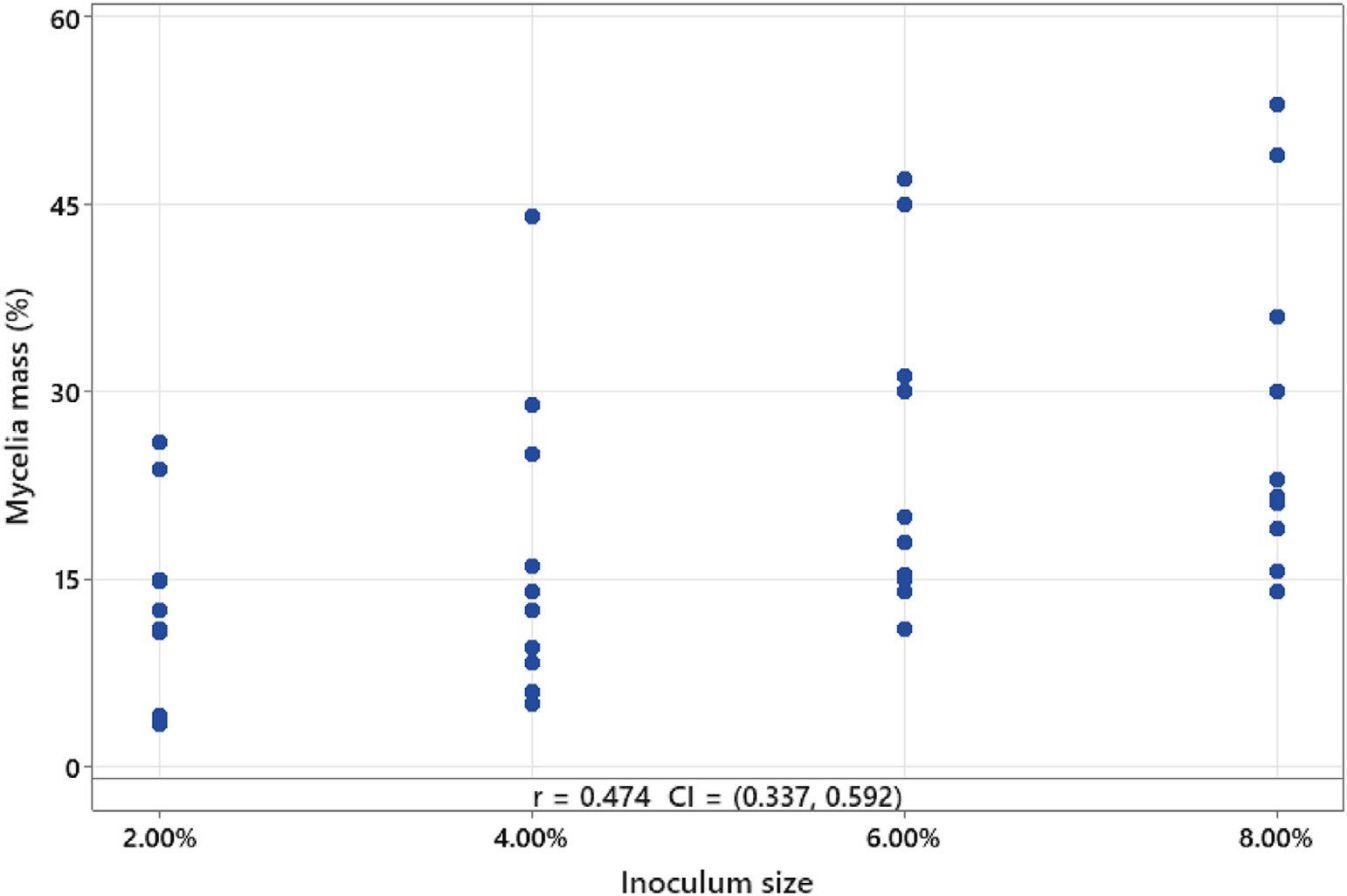
Matrix plot of inoculum size and mycelia mass (%), 95% confidence level for Pearson correlation.

The analysis of the relationship between inoculum size and mycelial biomass as shown in Figure 3 indicated a moderate positive correlation (*r* = 0.474), implying that as inoculum size increased, mycelial biomass production also tended to increase. The 95% confidence interval (CI = 0.337 to 0.592) confirms the statistical significance of this correlation, as the range does not include zero.

The distribution of data points shows variability in biomass production at different inoculum levels. While biomass generally increased with inoculum size, overlapping values suggest that additional factors, such as temperature and light intensity, may contribute to variations in fungal growth.

### 3.4 Effect of Temperature on Mycelial Biomass Production

**FIGURE 4:**
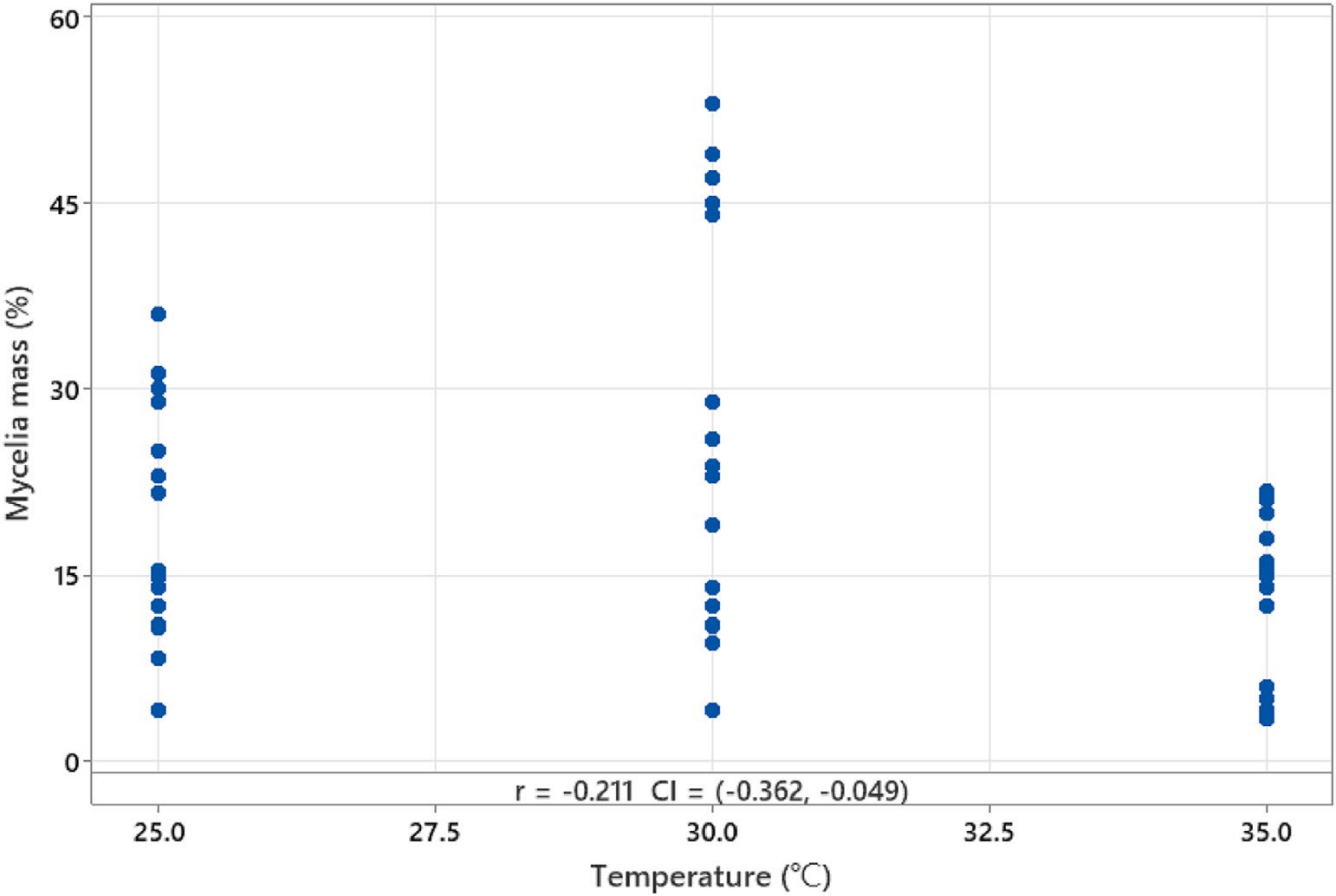
Matrix plot of temperature (°C) and mycelia mass (%), 95% confidence level for Pearson correlation.

The analysis of the relationship between temperature and mycelial biomass as shown in Figure 4 indicates a weak negative correlation (r = −0.211), implying that as temperature increased, mycelial biomass production showed a slight decline. The 95% confidence interval (CI = −0.362 to −0.049) confirms the statistical significance of this correlation, as the range does not include zero.

According to the biomass value distribution, the highest production took place at 30°C, whereas lower values were noted at 25°C and 35°C, respectively. This implies that mycelial growth might be at its best at 30°C, with growth possibly being inhibited by higher temperatures (35°C).

### 3.5 Effect of Light Conditions on Mycelial Biomass Production

**FIGURE 5:**
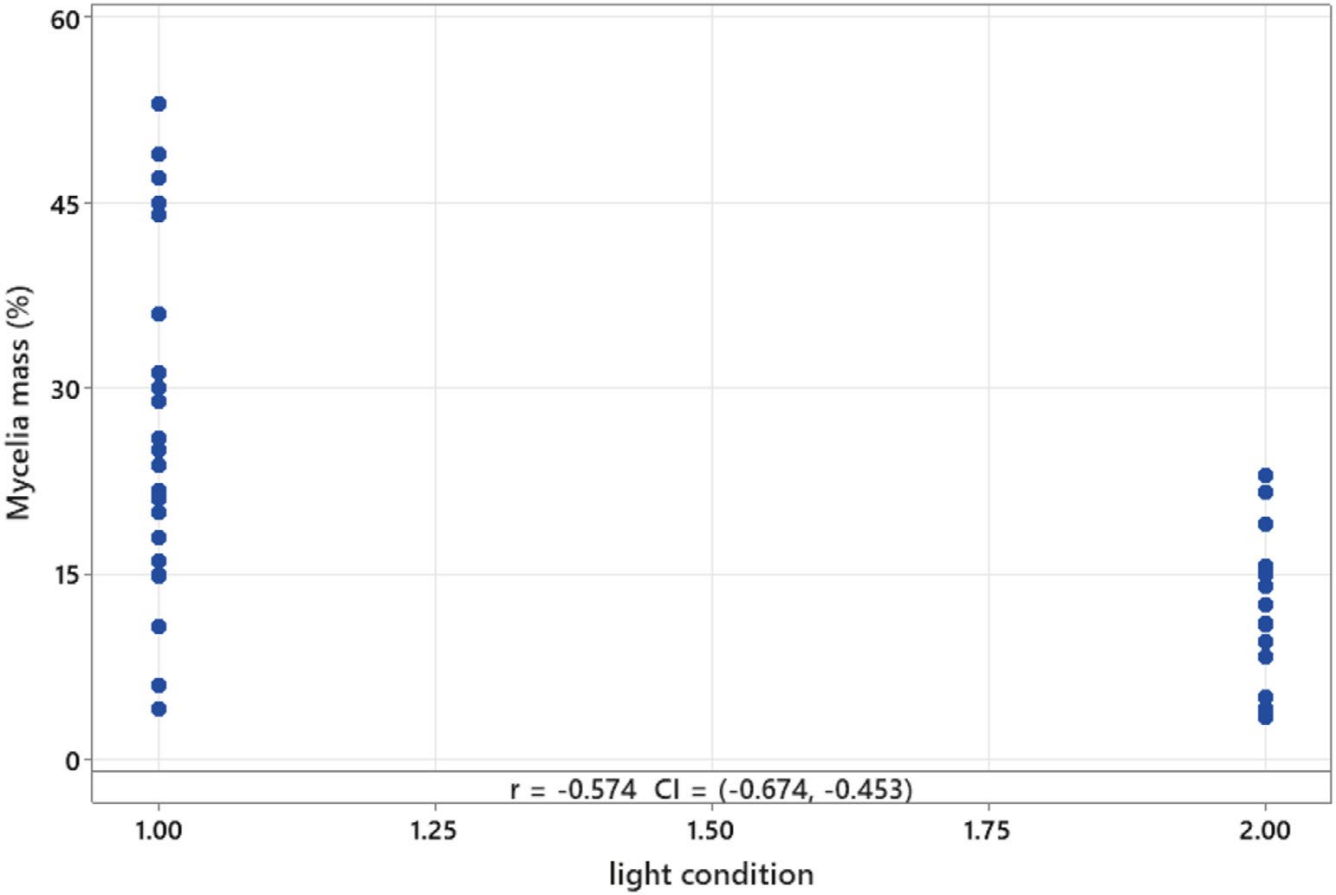
Matrix plot of light conditions (where 1 = darkness and 2 = 12 h light exposure) and mycelial biomass (%), showing Pearson correlation coefficients at the 95% confidence level.

Mycelial biomass decreased with increased light exposure, according to the analysis of the relationship between light conditions and mycelial biomass, which showed a moderately negative correlation (r = −0.574) (Figure 5). Since zero is not included in the range, the 95% confidence interval (CI = −0.674 to −0.453) validates the statistical significance of this correlation.

According to the biomass value distribution, mycelial growth was substantially greater in total darkness (shown as 1) than in the 12‐h light/12‐h dark cycle (shown as 2). This implies that for the best biomass accumulation, *Ganoderma* species prefer dark environments.

### 3.6 Proximate Composition of *Ganoderma enigmaticum* and *Ganoderma resinaceum*

The proximate composition of the two species is presented in Table 4. Moisture content was significantly higher (*p* < 0.05) in *G. resinaceum* (48.8–50.0 g/100 g) compared to *G. enigmaticum* (43.6–43.8 g/100 g). Ash content ranged from 0.96% to 1.62%, with no significant differences (*p* > 0.05) between species. Fat content was significantly higher (*p* < 0.05) in *G. enigmaticum* (2.16 ± 0.01 g/100 g) compared to *G. resinaceum* (0.92–1.14 g/100 g). Protein content varied between 9.65–10.7 g/100 g, with *G. enigmaticum* recording the highest values, but the difference was not statistically significant (*p* = 0.089). The crude fiber was significantly higher (*p* < 0.05) in *G. enigmaticum* (7.80–8.11 g/100 g) compared to *G. resinaceum* (2.86–4.42 g/100 g). Carbohydrate content was also significantly higher (*p* < 0.05) in *G. enigmaticum* (42.3–43.6 g/100 g) compared to *G. resinaceum* (38.0–38.6 g/100 g).

**TABLE 4:** Mean proximate analysis of *Ganoderma enigmaticum* and *Ganoderma resinaceum*.

Samples	Moisture (g/100 g)	Ash (g/100 g)	Fat (g/100 g)	Protein (g/100 g)	Crude Fiber (g/100 g)	Carbohydrate (g/100 g)
GE 254D	43.7 ± 0.47^C^	1.04 ± 0.01^AB^	1.70 ± 0.10^B^	9.99 ± 0.50^bc^	7.81 ± 0.05^B^	43.6 ± 1.06^A^
GE 256D	43.7 ± 0.36^C^	0.96 ± 0.02^B^	1.70 ± 0.14^B^	10.3 ± 0.10^AB^	7.81 ± 0.04^B^	43.3 ± 0.39^A^
GE 258D	43.8 ± 0.08^C^	1.06 ± 0.02^AB^	2.16 ± 0.01^A^	10.7 ± 0.03^A^	8.11 ± 0.03^A^	42.3 ± 0.09^A^
GE 302D	43.7 ± 0.44^C^	1.62 ± 0.58^A^	1.70 ± 0.10^B^	9.65 ± 0.48^C^	7.80 ± 0.04^B^	43.3 ± 1.47^A^
GE 304D	43.6 ± 0.45^C^	1.04 ± 0.01^AB^	1.70 ± 0.15^B^	10.3 ± 0.10^AB^	7.81 ± 0.04^B^	43.3 ± 0.48^A^
GE 306D	43.7 ± 0.12^C^	1.06 ± 0.01^AB^	2.16 ± 0.01^A^	10.6 ± 0.03^A^	8.11 ± 0.03^A^	42.3 ± 0.14^A^
GE 308D	43.7 ± 0.08^C^	1.06 ± 0.01^AB^	2.18 ± 0.01^A^	10.7 ± 0.02^A^	8.11 ± 0.02^A^	42.3 ± 0.09^A^
GR 254D	49.4 ± 0.17^AB^	1.02 ± 0.01^AB^	0.92 ± 0.05^C^	9.99 ± 0.02^bc^	2.87 ± 0.07^D^	38.6 ± 0.23^B^
GR 256D	49.9 ± 0.17^AB^	1.05 ± 0.04^AB^	0.94 ± 0.05^C^	10.0 ± 0.06^ABC^	2.86 ± 0.05^D^	38.0 ± 0.06^B^
GR 258D	49.8 ± 0.18^A^	1.05 ± 0.02^AB^	0.96 ± 0.03^C^	10.0 ± 0.03^bc^	2.88 ± 0.06^D^	38.2 ± 0.25^B^
GR 304D	49.6 ± 0.17^AB^	1.03 ± 0.01^AB^	0.95 ± 0.04^C^	10.0 ± 0.06^ABC^	2.87 ± 0.06^D^	38.4 ± 0.26^B^
GR 306D	50.0 ± 0.03^A^	1.05 ± 0.04^AB^	0.93 ± 0.05^C^	10.0 ± 0.06^ABC^	2.86 ± 0.05^D^	38.0 ± 0.06^B^
GR 308D	48.8 ± 0.49^B^	1.11 ± 0.01^AB^	1.14 ± 0.01^C^	10.5 ± 0.10^AB^	4.42 ± 0.01^C^	38.4 ± 0.58^B^

*Note:* Data was reported as means ± standard deviation of proximate composition of *Ganoderma enigmaticum* and *Ganoderma resinaceum* at different growth conditions. Means in column that do not share the same letter are significantly different (*p* ≤ 0.05). Analysis was performed in triplicates.

### 3.7 Antioxidant Activity of *Ganoderma enigmaticum* and *Ganoderma resinaceum*

The results of antioxidant activities of *G. resinaceum* and *G. enigmaticum* are indicated in Table 5. The lowest EC_50_ value (highest antioxidant activity) was observed in *G. resinaceum* (GR308D) at 6.7 ± 0.10 mg/mL, followed by GR256D (7.2 ± 0.10 mg/mL) and GE308D (7.4 ± 0.10 mg/mL). The highest EC_50_ value (weakest antioxidant activity) was recorded in *G. enigmaticum* (GE302D) at 13.6 ± 0.11 mg/mL, which was significantly different from all other treatments (*p* < 0.05).

**TABLE 5:** Mean antioxidant activity (EC_50_) of *Ganoderma enigmaticum* and *Ganoderma resinaceum*.

Samples	EC_50_ (mg/mL)
Ascorbic acid (control)	0.01 ± 0.20^H^
GE 254D	13.4 ± 0.10^A^
GE 256D	10.5 ± 0.15^B^
GE 258D	9.5 ± 0.10^C^
GE 302D	13.6 ± 0.11^A^
GE 304D	7.8 ± 0.05^E^
GE 306D	7.6 ± 0.11^E^
GE 308D	7.4 ± 0.10^C^
GR 254D	9.2 ± 0.10^C^
GR 256D	7.2 ± 0.10^F^
GR 258D	7.6 ± 0.12^E^
GR 304D	8.6 ± 0.10^D^
GR 306D	8.7 ± 0.01^CD^
GR 308D	6.7 ± 0.10^G^

*Note:* Data was reported as means ± standard deviation of EC_50_ of *Ganoderma enigmaticum* and *Ganoderma resinaceum* at different growth conditions. Means in column that do not share the same letter are significantly different (*p* ≤ 0.05). Analysis was performed in triplicates.

### 3.8 Mineral Analysis of *Ganoderma enigmaticum* and *Ganoderma resinaceum* Under Different Growth Conditions

The mineral composition of *G. resinaceum* and *G. enigmaticum* is shown in Table 6. The phosphorus content varied significantly (*p* < 0.05), with *G. enigmaticum* having the highest level (941 mg/kg). *G. resinaceum* had substantially higher potassium levels (472 mg/kg) (*p* = 0.031). Calcium and sulfur levels varied significantly with temperature and inoculum size (*p* < 0.05), with the highest levels observed at 30°C with an 8% inoculum size.

**TABLE 6:** Mean concentrations of essential minerals in *Ganoderma enigmaticum* and *Ganoderma resinaceum*.

Samples	Potassium (K) (mg/kg)	Phosphorus (P) (mg/kg)	Magnesium (Mg) (mg/kg)	Calcium (Ca) (mg/kg)	Sulfur (S) (mg/kg)
GE 254D	222.33 ± 5.77^D^	641.67 ± 7.51 ^F^	3349.00 ± 29.00 ^D^	730.00 ± 9.00 ^B^	148.00 ± 18.00 ^B^
GE 256D	293.00 ± 4.58 ^C^	666.67 ± 10.50 ^F^	3428.00 ± 38.00 ^D^	744.00 ± 11.00 ^B^	154.00 ± 18.00 ^B^
GE 258D	421.00 ± 31.00 ^AB^	743.90 ± 7.15 ^E^	3412.00 ± 27.00 ^D^	795.67 ± 18.50 ^B^	160.00 ± 17.00 ^B^
GE 302D	400.00 ± 26.00^B^	881.67 ± 7.51 ^BCD^	3792.00 ± 34.00 ^B^	815.00 ± 12.00 ^bcD^	180.00 ± 15.00 ^AB^
GE 304D	412.00 ± 3.00 ^AB^	917.67 ± 7.51 ^AB^	3676.00 ± 35.00 ^BC^	870.00 ± 15.00 ^B^	192.00 ± 14.00 ^AB^
GE 306D	407.00 ± 7.00 ^B^	923.67 ± 8.50 ^AB^	3622.00 ± 35.00 ^C^	859.00 ± 13.00 ^B^	183.00 ± 14.00 ^AB^
GE 308D	414.00 ± 13.00 ^AB^	940.67 ± 21.50 ^A^	3815.00 ± 38.00 ^B^	884.00 ± 16.00 ^B^	209.00 ± 24.00 ^A^
GR 254D	472.00 ± 17.00 ^A^	843.00 ± 20.00 ^D^	3679.00 ± 55.00 ^BC^	732.00 ± 11.00 ^B^	149.00 ± 14.00 ^B^
GR 256D	460.00 ± 21.00 ^AB^	859.00 ± 12.00 ^CD^	3628.00 ± 46.00 ^C^	730.00 ± 11.00 ^B^	149.00 ± 15.00 ^B^
GR 258D	438.00 ± 34.00 ^AB^	875.00 ± 20.00 ^BCD^	3598.00 ± 72.00 ^C^	755.00 ± 14.00 ^B^	157.00 ± 14.00 ^B^
GR 304D	461.00 ± 26.00 ^AB^	906.00 ± 43.00 ^ABC^	3791.00 ± 32.00 ^B^	803.00 ± 15.00 ^B^	179.00 ± 15.00 ^AB^
GR 306D	437.00 ± 28.00 ^AB^	910.00 ± 0.40 ^ABC^	3976.00 ± 62.00 ^A^	844.00 ± 16.00 ^B^	183.00 ± 14.00 ^AB^
GR 308D	431.00 ± 22.00 ^AB^	916.00 ± 27.00 ^AB^	4004.00 ± 92.00 ^A^	851.00 ± 16.00 ^B^	213.00 ± 15.00 ^A^

*Note:* Data are reported as mean ± standard deviation for the essential mineral concentrations of *Ganoderma* enigmaticum and *Ganoderma resinaceum* under different growth conditions. Within each column, means with different superscript letters are significantly different at *p* < 0.05, whereas means sharing at least one superscript letter are not significantly different.

## 4 Discussion

This study demonstrated that mycelial growth and biomass accumulation in *G. resinaceum* and *G. enigmaticum* were significantly influenced by temperature, light conditions, and inoculum size. Because the samples completed myceliating the earliest and had the highest mycelial lengths, the observed results validate that 30°C is the ideal temperature for mycelial growth.

However, a temperature of 25°C resulted in a slight delay with still substantial growth, indicating that this temperature is also suitable, although less efficient than the 30°C. This result is in line with earlier research that found that *Ganoderma* species exhibit increased metabolic efficiency and enzymatic activity at 25°C–30°C, which promotes quick substrate colonization (Jayasinghe et al. 2008; Du et al. 2019). Growth suppression at 35°C is consistent with data showing that high temperatures impair fungal viability by causing protein denaturation, membrane instability, and oxidative stress (Chatterjee and Sengupta 2016; Yousefi and Abbasi 2022).

The idea that *Ganoderma* species can withstand a wide range of temperatures but perform best within a specific window is further supported by the weak negative correlation between temperature and mycelial biomass. Despite some apparent growth, higher temperatures may cause metabolic stress, which would reduce biomass accumulation. Fungal biomass has been shown to vary with temperature, and species‐specific responses are impacted by structural and enzymatic stability (Paterson 2006; Kuvarina et al. 2022). Therefore, it seems that in controlled cultivation systems, maintaining an ideal temperature of about 30°C is crucial to achieving the best biomass yield.

A strong positive correlation was also observed between inoculum size and mycelial expansion. High inoculum percentages (6%–8%) resulted in faster substrate colonization. This was likely due to reduced lag phase and increased fungal biomass availability. These conditions also resulted in earlier visible complete mycelial colonization in treatments receiving higher inoculum treatments. The finding aligns with studies indicating that larger fungal biomass at inoculation shortens the lag phase and accelerates substrate colonization (Hickman 2015; van Kuijk et al. 2016). In contrast, lower inoculum sizes (2% and 4%) exhibited prolonged growth phases, with strains such as GE252D, GE254L, GR252D, and GR254L completing their myceliation only between days 10 and 14.

Although most mycelial lengths equalized across all inoculum sizes in high‐growth samples by the final time point, biomass accumulation remained significantly different, with larger inoculum sizes yielding greater mycelial mass. This highlights the role of mycelial density over linear extension in determining total biomass yield, a pattern previously reported in both edible and medicinal mushrooms (Chang and Miles 2004; Nasreen et al. 2005). The significant confidence interval (CI = 0.337 to 0.592) and moderate positive correlation (*r* = 0.474) highlight the significance of optimizing the inoculum to maximize biomass production. However, they also suggest that other environmental factors, like substrate quality and incubation conditions, may affect the final yield.

Light conditions also exerted a significant effect on mycelial development. Mycelial growth in darkness was consistently higher than in light exposed samples, indicating that light exposure slows down mycelial colonization and reduces overall biomass accumulation. This supports reports that darkness promotes fungal vegetative growth by minimizing photoreceptor activation, thereby conserving energy for hyphal elongation (Wang et al. 2011; Muangkram et al. 2023). The moderate negative correlation between light exposure and mycelial biomass (r = −0.574; CI = −0.674 to −0.453) further underscores this relationship, demonstrating that light exposure slows down mycelial colonization and reduces overall biomass accumulation.

Nevertheless, the ability of light‐exposed samples to still achieve substantial growth suggests some level of adaptability within *Ganoderma* species, making it possible to cultivate them under various lighting conditions when necessary. Light, known to influence fungal metabolism, sporulation, and secondary metabolite production, may redirect metabolic resources toward reproductive structures at the expense of vegetative expansion (Paterson 2006; Boh et al. 2007; Xie et al. 2012). Therefore, cultivation strategies that maximize biomass rather than spore production should prioritize minimal light exposure or complete darkness, especially during early growth.

Interestingly, despite mycelial length maximizing at 8.70 mm by day 12 in rapid‐growth samples (GE308D, GE306D, GE258D, GR308D, GR306D, and GR258D), mycelial mass continued to exhibit variations across temperature, light, and inoculum size conditions. This discrepancy suggests that while linear growth reached a limit, biomass accumulation was still influenced by metabolic efficiency, nutrient uptake, and hyphal density. Such observations have been previously reported, where biomass accumulation in Ganoderma species depends not only on hyphal elongation but also on internal structural development, including hyphal branching patterns and density (Paterson 2006; Gryzenhout et al. 2021).

The proximate composition analysis showed significant differences between *G. resinaceum* and *G. enigmaticum*. *G. enigmaticum* exhibited higher crude fiber, carbohydrate, and fat content, while *G. resinaceum* had higher moisture retention. These findings is in line with previous studies that reported species‐specific differences in fungal cell wall composition, lipid biosynthesis, and carbohydrate metabolism (Paudel et al. 2016; Wang et al. 2017). The higher fiber content of G. enigmaticum indicates its potential application in functional foods as a dietary fiber source, while the higher moisture content of G. resinaceum may influence its textural properties and shelf stability.

The differences in antioxidant potential observed between *G. enigmaticum and G. resinaceum* align with previous findings that report species‐dependent variations in bioactive compound production (Hamwenye et al. 2022). *Ganoderma* species contain bioactive polysaccharides, flavonoids, and triterpenes, all contributing to their radical‐scavenging properties. The enhanced antioxidant activity observed in GR308D and GR256D suggests that *G. resinaceum* strains incubated at 30°C and 25°C with an 8% and 6% inoculum size, respectively, supported the biosynthesis of antioxidant compounds.

Temperature has been widely reported as a critical factor affecting fungal metabolism and secondary metabolite production Similarly, inoculum size has been shown to influence biomass production, and metabolite synthesis, often yielding enhanced bioactive compound production (Nasreen et al. 2005; Hickman 2015; van Kuijk et al. 2015).

The variability in EC_50_ values among *Ganoderma* strains could serve as valuable candidates for nutraceutical development, functional foods, and antioxidant‐based therapeutic products. The higher antioxidant potential observed in *G. resinaceum* strains compared to *G. enigmaticum* suggests that species‐specific metabolic pathways contribute to differences in antioxidant efficiency. Although there aren't many studies specifically comparing *Ganoderma resinaceum's* triterpenoid and polysaccharide content to those of other *Ganoderma* species, what is known suggests that the bioactive chemical profiles of various *Ganoderma* species differ significantly. For example, a study comparing *Ganoderma lucidum* and *Ganoderma sinense* revealed that although their polysaccharide profiles were comparatively similar, the two species' triterpene contents differed significantly (Xie et al. 2012).

The high antioxidant properties as observed in GR308D, GR256D, and GE304D indicate that these strains could serve as valuable candidates for nutraceutical development, functional foods, and antioxidant‐based therapeutic products. Potent free radical scavenging activity suggests potential applications in preventing oxidative stress‐related disorders, including cardiovascular diseases, neurodegenerative conditions, and metabolic syndromes (Boh et al. 2007).

According to the results, the species type, incubation temperature, and inoculum size all have a substantial impact on the antioxidant activity of *G. enigmaticum* and *G. resinaceum*. Samples of *G. resinaceum*, especially GR308D, showed the highest antioxidant activity, while samples of *G. enigmaticum*, including GE302D and GE254D, showed the lowest activity. These results demonstrate how environmental and genetic factors affect Ganoderma species' antioxidant capability.

According to the mineral composition analysis, *G. resinaceum* had more potassium while *G. enigmaticum* had more phosphorus. This is consistent with research by Sanmee et al. (2003), which found that wild mushrooms have significant quantities of potassium and phosphorus. The researchers found that the potassium levels ranged from 12.8 to 45.2 mg/g dry weight, while the phosphorus levels were between 2.1 and 8.1 mg/g dry weight. *G. enigmaticum's* high phosphorus content would indicate temperature‐dependent phosphorus absorption. Given potassium's critical role in preserving cardiovascular stability and muscle contraction, the greater potassium concentration in *G. resinaceum* raises the possibility of usage in potassium‐enriched food formulations (Gums 2004; Turban et al. 2021).

The findings also demonstrated that inoculum size and temperature had a substantial impact on mineral accumulation, with increasing phosphorus, calcium, and sulfur uptake at 30°C and at larger inoculum sizes (6% and 8%). This is in line with research that indicates that ideal substrate and temperature conditions increase the efficiency of fungal nutrient absorption, which raises the amount of minerals retained in the biomass (Okada and Ferris 2001).

## 5 Conclusion

The findings from this study demonstrate that temperature, light exposure, and inoculum size significantly influence the mycelial growth, biomass yield, and biochemical composition of *Ganoderma enigmaticum* and *Ganoderma resinaceum*. Optimal growth was achieved at 30°C with 6%–8% inoculum size under dark conditions. These conditions promoted biomass production, mineral accumulation (especially phosphorus, calcium, and sulfur), and accelerated mycelial colonization. Nutritionally, *G. enigmaticum* exhibited higher fiber, fat, and carbohydrate content, making it a promising candidate for dietary and functional food formulations. In contrast, *G. resinaceum* showed superior antioxidant activity and higher potassium content, suggesting potential for potassium‐enriched functional food applications. The study confirms that genetic and environmental factors contribute to the functional qualities of these mushrooms. Thus, tailored cultivation strategies can be employed to enhance their use in the health and wellness industry.

## Author Contributions

**Matilda Dzomeku:** visualization, writing – review and editing, resources. **Gideon Adotey:** writing – review and editing, visualization, resources. **Florence Adwoa Ayirezang:** conceptualization, methodology, data curation, investigation, formal analysis, resources, writing – original draft, writing – review and editing. **Ibok Nsa Oduro:** supervision, visualization, writing – review and editing, validation. **William Otoo Ellis:** supervision, conceptualization, writing – review and editing, validation, visualization, formal analysis.

## Funding

The authors have nothing to report.

## Ethics Statement

This study did not involve human participants or animals and therefore did not require ethics approval.

## Consent

This study did not involve human participants and therefore informed consent was not applicable.

## Conflicts of Interest

The authors declare no conflicts of interest.

## Data Availability

The data that support the findings of this study are available from the corresponding author upon reasonable request.
